# Pathophysiological characterization of asthma transitions across adolescence

**DOI:** 10.1186/s12931-014-0153-7

**Published:** 2014-11-29

**Authors:** Syed Hasan Arshad, Abid Raza, Laurie Lau, Khalid Bawakid, Wilfried Karmaus, Hongmei Zhang, Susan Ewart, Veersh Patil, Graham Roberts, Ramesh Kurukulaaratchy

**Affiliations:** The David Hide Asthma and Allergy Research Centre, Isle of Wight, UK; Clinical and Experimental Sciences, Faculty of Medicine, University of Southampton, Southampton, UK; Respiratory Biomedical Research Unit, University Hospital Southampton, Tremona Road, Southampton, UK; Division of Epidemiology, Biostatistics, and Environmental Health, School of Public Health, University of Memphis, Memphis, TN USA; Department of Large Animal Clinical Sciences, Michigan State University, East Lansing, MI USA

## Abstract

**Background:**

Adolescence is a period of change, which coincides with disease remission in a significant proportion of subjects with childhood asthma. There is incomplete understanding of the changing characteristics underlying different adolescent asthma transitions. We undertook pathophysiological characterization of transitional adolescent asthma phenotypes in a longitudinal birth cohort.

**Methods:**

The Isle of Wight Birth Cohort (N = 1456) was reviewed at 1, 2, 4, 10 and 18-years. Characterization included questionnaires, skin tests, spirometry, exhaled nitric oxide, bronchial challenge and (in a subset of 100 at 18-years) induced sputum. Asthma groups were “never asthma” (no asthma since birth), “persistent asthma” (asthma at age 10 and 18), “remission asthma” (asthma at age 10 but not at 18) and “adolescent-onset asthma” (asthma at age 18 but not at age 10).

**Results:**

Participants whose asthma remitted during adolescence had lower bronchial reactivity (odds ratio (OR) 0.30; CI 0.10 -0.90; p = 0.03) at age 10 plus greater improvement in lung function (forced expiratory flow 25-75% gain: 1.7 L; 1.0-2.9; p = 0.04) compared to persistent asthma by age 18. Male sex (0.3; 0.1-0.7; p < 0.01) and lower acetaminophen use (0.4; 0.2-0.8; p < 0.01) independently favoured asthma remission, when compared to persistent asthma. Asthma remission had a lower total sputum cell count compared to never asthma (31.5 [25–75 centiles] 12.9-40.4) vs. 47.0 (19.5-181.3); p = 0.03). Sputum examination in adolescent-onset asthma showed eosinophilic airway inflammation (3.0%, 0.7-6.6), not seen in persistent asthma (1.0%, 0–3.9), while remission group had the lowest sputum eosinophil count (0.3%, 0–1.4) and lowest eosinophils/neutrophils ratio of 0.0 (Interquartile range: 0.1).

**Conclusion:**

Asthma remission during adolescence is associated with lower initial BHR and greater gain in small airways function, while adolescent-onset asthma is primarily eosinophilic.

**Electronic supplementary material:**

The online version of this article (doi:10.1186/s12931-014-0153-7) contains supplementary material, which is available to authorized users.

## Introduction

Remission, and occasionally relapse, of symptoms is observed in many asthmatics over the life course [1-4]. Adolescence is a period of change that is associated with puberty and rapid physical growth. Clinical remission of asthma is highest during this period [1], and typically defined as those with a previous diagnosis of asthma who are asymptomatic at a subsequent assessment without the need for treatment for a period of at least 12 months [2-4]. The rate of asthma remission during adolescence has been variably reported to range from 30 to 65% [4-7].

Individuals with clinical asthma remission have been shown to have persistent subclinical disease, characterized by low lung function, bronchial hyper-responsiveness (BHR), airway inflammation or remodeling [2,8,9]. However, characteristics of airway inflammation might be different in those with remittent compared to persistent asthma. Moreover, changes and risk factors during adolescence that lead to remission of symptoms remain unclear.

There is another group of children who acquire asthma for the first time at this age. In the Tucson birth cohort, 27% (49 of 181) of asthma at 22 years developed after 16 years of age, although some had wheezed during early childhood [10]. We have recently reported that in the Isle of Wight birth cohort, 28% of asthma at age 18 was of adolescent-onset [11]. Both puberty and lung growth have been suggested as factors determining asthma outcome during adolescence [5,6,12], which may be associated with variable immune response resulting in different types or degree of airway inflammation.

Asthma can be regarded as a diverse syndrome with varying clinical and pathophysiological characteristics that may influence disease natural history and outcome during adolescence. Our overall hypothesis was that significant and distinct changes in the pathophysiology of asthma occur during remission of existing asthma or development of new asthma during adolescence. The primary aim was to investigate the pathophysiological characteristics associated with adolescent asthma transitions. Study of these characteristics and risk factors would highlight important differences between these groups and provide insights into the management and prevention of asthma during that critical phase in life. Using data and samples from the Isle of Wight birth cohort, we explored the “changing face” of asthma during adolescence.

### Research questions

1. What are the pathophysiological characteristics associated with persistence of asthma during adolescence? To investigate this, we compared asthma remission with persistent asthma.
2. What are the factors associated with asthma remission? To investigate this we tested early life and adolescent factors relevant to asthma.
3. What are the residual pathophysiological abnormalities in remittent asthmatics while symptoms have improved? To investigate this, we compared asthma remission with never asthma at age 18 years.
4. What are the pathophysiological characteristics of asthma that develops during adolescence? To investigate this, we compared adolescent-onset asthma with never asthma and persistent asthma.

The Isle of Wight cohort, with its population-based prospective design and extensive phenotyping over the entire childhood period provides a powerful means of approaching these questions.

## Materials and methods

A whole population birth cohort was established on the Isle of Wight in 1989 (n = 1456). These children have been followed at the ages of 1, 2, 4, 10 and 18 years [13-18]. All participants provided informed consent and ethical approval was obtained from the Isle of Wight, Portsmouth and Hampshire Local Research Ethics Committee (06/Q1701/34). At 10 and 18 years, validated questionnaires were completed from face to face interview (those who attended the Centre) or telephone or postal questionnaires (Additional file 1: Table S1). Participants attending the Centre in person also underwent spirometry, assessment of bronchial hyper-responsiveness (BHR), fractional exhaled nitric oxide (FeNO) measurement and skin prick test (SPT). Details of questionnaires and methods for SPT, spirometry, BHR and FeNO have been reported previously [11,17,18].

Briefly, both study-specific and International Study of Asthma and Allergies in Childhood questionnaires were completed for detailed assessment of asthma symptoms and its treatment. Information on environmental risk factors was collected from birth up to age 18 years such as birth weight, method of feeding, current and past cigarette smoking, pets and socio-economic class. At 18-years, study participants reported average monthly use of acetaminophen and NSAID (non-steroidal anti-inflammatory drugs) during the past year.

For spirometry, American Thoracic Society (ATS) guidelines were followed to ensure validity and reproducibility using the Koko system (Koko Spirometer Longmont, CO, USA) [19]. To adjust for sex and height effect on FEV_1_, FVC, and FEF_25–75_, we regressed these variables on sex and height; the residuals with sex and height effect excluded were used in the analyses. We used this approach in preference to using % predicted lung function values. With % predicted values, people with the same sex and height would register the same value of % predicted estimated from existing formulas based on reference populations. Those reference ranges may not be applicable to our population and would not necessarily reflect the measure of lung function for each individual participant in our cohort. Methacholine bronchial challenge was performed using the protocol recommended by the ATS [20]. To perform spirometry or bronchial challenge, participant were required to be free from respiratory infection for 14 days, not taking oral steroids, not taken beta_2_ agonist for 6 hours and abstained from caffeine intake for at least 4 hours. BHR was determined by methacholine concentration causing a 20% fall in FEV_1_ from the post-saline value, expressed as PC_20_ with a positive test defined by PC_20_ < 8 mg/ml. A continuous dose–response slope (DRS) measure of BHR was estimated by least-square regression of percentage change in FEV_1_ upon each successive incremental dose of methacholine administered for each child. A transformation of Log10 (DRS+ 10) was required to satisfy the distributional assumption of normal data with higher positive values signifying greater bronchial reactivity. We used a continuous dose–response measure of BHR since not all subjects who undergo bronchial provocation testing will demonstrate a 20% fall in FEV_1_ that enables calculation of a PC_20_ to indicate BHR. Reliance on PC_20_ would have meant that a proportion of subjects would not provide meaningful data on BHR. FeNO was measured using Niox mino (Aerocrine AB, Solna, Sweden) according to ATS recommendations [21]. Measurements were made before spirometry testing with the subject standing without a nose clip. SPT was performed to a panel of common food/aeroallergens (house dust mite, grass pollen mix, tree pollen mix, cat, dog, *Alternaria alternata*, *Cladosporium herbarum*, milk, hens’ egg, wheat, soya, cod and peanut) using allergen extract from ALK-Abello (Horsholm, Denmark). SPT was regarded as positive when the mean of the largest diameter plus its perpendicular was at least 3 mm larger than the negative control. Subjects with one or more positive SPT were regarded as atopic.

The 18 year follow-up for the birth cohort was done in two stages. In phase 1, subjects completed questionnaires and had skin test and lung function test during the first visit (as described above). The working definition of asthma at both 10 and 18 years was “physician diagnosed asthma” and “wheezing or asthma treatment in the last 12 months”. We then identified subjects who belonged to the four adolescent asthma groups based on the following criteria. “Remission” was defined as having asthma at age 10 but no asthma at age 18 years (absence of asthma symptoms or medication for 12 months). “Persistence” was defined as having asthma at both 10 and 18 years. Adolescent-onset asthma was defined as asthma at age 18 but not at age 10 years. The reference group of “never asthma” consisted of participants who did not report wheeze or were given a diagnosis of asthma at any follow-up (1, 2, 4, 10 and 18 years). Thus, participants who reported wheeze or asthma at 1, 2 and 4 years but lost symptoms at age 10 were excluded from the current analysis. In phase 2, from each group (asthma remission, persistent asthma, adolescent-onset asthma and never asthma), subjects were randomly selected to undergo sputum induction (n = 100). Subjects from each group were invited to attend in sequence, according to their study number, for a second visit when sputum induction was performed using a standard protocol [22]. We stopped, once the required number of around 20 in remittent asthma and adolescent onset asthma groups and around 30 in persistent and never asthma were reached (the latter 2 groups being larger in size then the former two groups). Following baseline spirometry, 400 μg salbutamol was administered with spirometry repeated after 10 minutes. If FEV1 was ≥60% predicted, participants received serial nebulization of hypertonic saline (4.5%) for 5 minutes up to a maximum of 20 minutes (5 × 4). Sputum samples were collected and immediately placed on ice and processed within 2 hours. Sputum plugs and obvious mucoid components were selected from the expectorate and weighed. 22.5 μL of protease inhibitor per gram of sputum and DTT was added at 4 times the weight of sputum. The sample was then placed on a cell rocker for 45 minutes before being sieved through a sterile nylon mesh. This was then centrifuged for 10 minutes (1500 rpm). The supernatant was then separated and frozen at −80°C. Total and differential cell counts (epithelial cells, lymphocytes, eosinophils, basophils, macrophages and neutrophils) were recorded. The cellular compositions are presented as a percent of inflammatory cell type (n) to that of total cell count (N). Eosinophilic cationic protein was measured using MESACUP ECP TEST ELISA kit (MBL, CAltag-Medsystem, Buckinghamshire, UK) following manufacturer’s instructions.

### Statistical analysis

Data were entered into SPSS version 17 (IBM statistics). Categorical variables were assessed using Pearson’s chi-square tests. For continuous measures which passed the test of normality with or without transformation, we report mean values and independent samples t tests were applied for comparison. For variables demonstrating non-normal distributions such as sputum differential count, median values (with 25th and 75th centiles) are reported and compared using Mann–Whitney *U* test. A multivariate logistic regression model was employed to assess independent significance of these factors. All variables showing a trend for significance (p < 0.1) in univariate tests were included in the multivariate model. Two multiple logistic regression models were created, one using predictive factors only (present from birth until age 10 years) and a final model using all factors that showed suggestive associations (p < 0.1) at univariate risk factor analysis. A p-value <0.05 was taken to indicate statistical significance.

## Results

At 10 years, 94.3% (1373/1456) and at 18 years, 90.2% (1313/1456) of the cohort subjects were assessed with questionnaires. Information on asthma status at both 10 and 18-years was available for 1234 of 1456 (84.8%). Approximately 70% and 60% participants attended the Research Centre at 10 and 18 years respectively for full assessments including SPT, spirometry and methacholine bronchial challenge (Additional file 1: Table S1). Those attending the Centre for full data collection at 10 and 18 years did not differ in key characteristics from the overall study population [11,18].

Clinical remission during adolescence occurred in 31.0% (56/181) of subjects with asthma at the age of 10 years, while the rest (125) had persistent asthma to age 18 years. Of participants with asthma at 18-years who also had data at 10-yrs, 36.9% (73/198) had adolescent-onset asthma. Of 100 participants who underwent sputum induction and processing at age 18 years, spirometry was available in 98, differential count in 76 and supernatant was available for measurement of ECP in 63 adolescents. Characteristics of subjects who had sputum induction in each asthma group were compared to the whole group to ensure that major characteristics were similar (Additional file 1: Table S2). Those who underwent sputum induction were not significantly different from the population from which they were drawn in various asthma-related characteristics. As an internal validation, we first compared atopic subjects with non-atopic and those with and without asthma to confirm their respective characteristics. As expected, atopic subjects had higher BHR and FeNO as well as sputum eosinophils, indicating the presence of underlying (eosinophilic) airway inflammation (Additional file 1: Table S3). Those with current asthma at age 18 years (persistent asthma and adolescent-onset asthma) had significantly lower FEV_1_ and higher BHR and FeNO than those without current asthma (asthma remission and never asthma). Eosinophil count and ECP were also significantly higher in asthmatics (Additional file 1: Table S4). Physical measures of height, weight and BMI were similar between groups over adolescence with and without gender stratification (data not shown).

**Figure 1:**
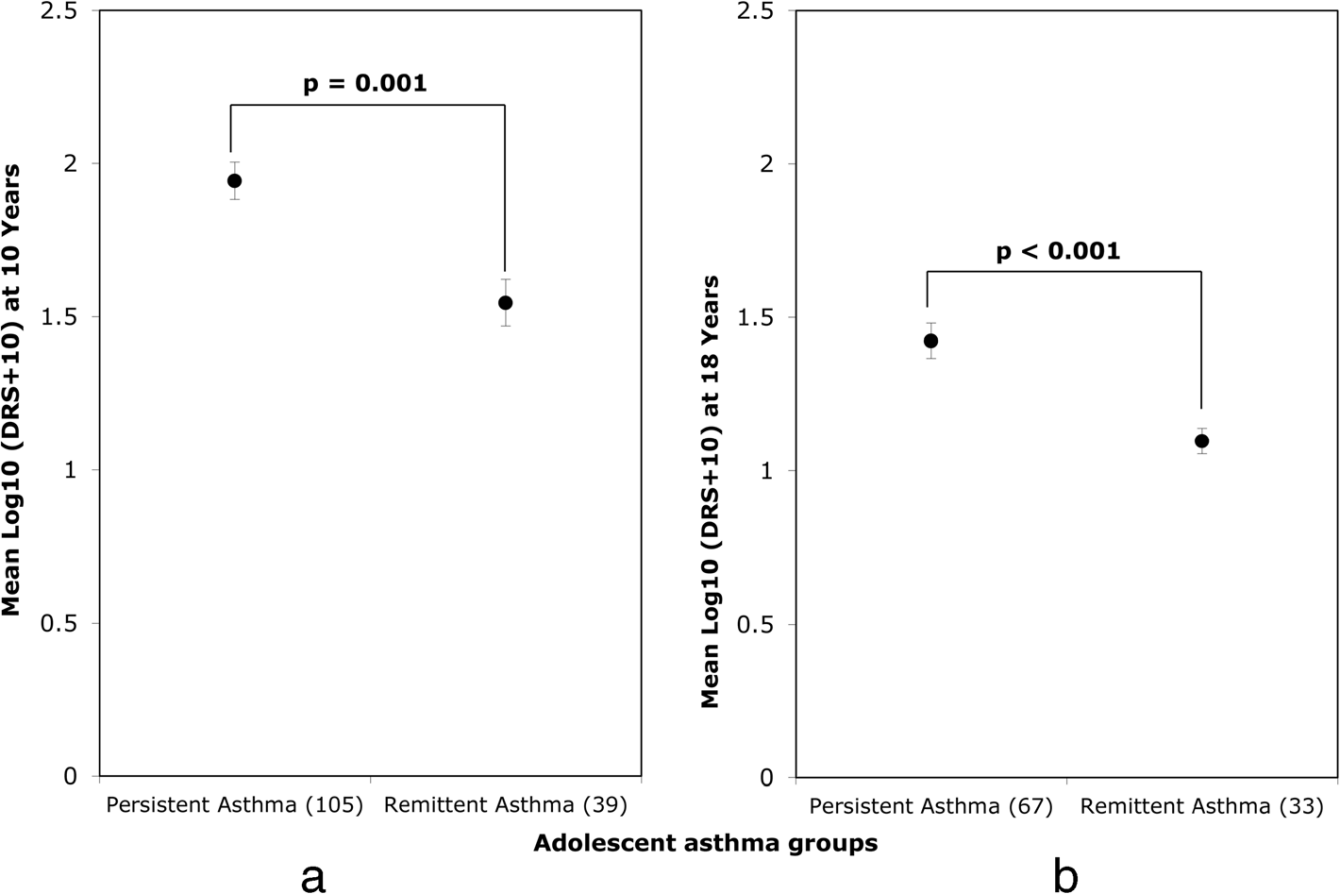
**Bronchial reactivity for adolescent asthma transition groups (remission versus persistent) at 10 and 18 years (a and b).** Children who go into remission during adolescence had lower bronchial hyperresponsiveness than persistent asthma at 10 years (when they still had asthma symptoms), as well as 18 years (when they had lost all symptoms). **a** (10 years). **b** (18 years).
  During adolescence: As adolescence is a period of growth and changes in lung function may occur differently in boys and girls, we stratified for sex, when analyzing spirometric data for changes during adolescence. Gain in lung function during adolescence (difference between 10 and 18 years) was higher in boys with asthma remission compared to persistent asthma for both FEV_1_ [2.6 L (SE: 0.1) versus 2.4 L (0.1); p: 0.04] and FEF_25–75_% [2.7 L (0.2) versus 2.1 L (0.1); p = 0.005). Similar improvements in pulmonary function of remittent females did not reach statistical significance (Table 2).

1. Comparison of asthma remission with persistent asthma: *to identify characteristics associated with persistence of asthma during adolescence*.
  At age 10 years: Asthma remission had less atopy (p = 0.01), current treatment (p = 0.002) and sleep disturbance (p = 0.04) than the persistent asthma (Table 1). Moreover, at this age, children destined for asthma remission had significantly lower BHR than those with persistent asthma (Figure 1a).

**Table 1:** **Asthma characteristics at age 10 years in adolescents whose asthma remitted or persisted between 10 and 18 years**

	**Remission % (n/N)**	**Persistent % (n/N)**	**Odds ratio**	**95% Confidence interval**	**p-value**
Frequent wheeze (>4/year)	38 (17/45)	50 (54/108)	0.6	0.3 – 1.2	0.17
Chest sounded wheezy with exercise	57 (32/56)	70 (87/125)	0.6	0.3 – 1.1	0.10
Sleep affected by wheeze	57 (28/49)	48 (52/108)	1.4	0.7 – 3.0	0.30
Sleep affected (≥1/week)	4 (2/49)	16 (17/108)	0.2	0.1 – 0.9	0.04
Wheezing severe enough to limit speech	13 (7/55)	15 (18/122)	0.8	0.3 – 2.1	0.72
Dry cough at night apart from a cold or chest infection	54 (30/56)	65 (80/124)	0.6	0.3 – 1.2	0.16
On current asthma treatment	76 (42/55)	93 (114/123)	0.2	0.1 – 0.6	0.002
On inhaled steroids at age 10	39.3 (22/56)	30.7 (35/114)	0.7	0.4 – 1.3	0.2
Atopy	43 (20/47)	70 (73/104)	0.4	0.2 – 0.8	0.01
Wheeze	89 (50/56)	88 (110/125)	1.1	0.4 – 3.1	0.80
Rhinitis	46 (25/55)	53 (64/121)	0.7	0.4 – 1.4	0.36

Notes:

Figures are percentage (numbers affected / total number). They refer to the previous year apart from atopy, which is defined as any positive skin prick test at 10 year assessment.

Comparisons in this table were made using Pearson’s chi-square test, with two sided significance set at p <0.05.

% (n/N) percentage, where n is the number of participants with the specific symptoms and N is the total number in that group.

**Table 2:** **Height adjusted pulmonary function: Gain in spirometric function from 10 to 18 years in those with asthma remission and persistence**

	**Remission**	**Persistent**	**Odds ratio**	**95% Confidence interval**	**p-value**
Male	n = 17	n = 40			
FEV_1_ (L) (S.E.)	2.6 (0.1)	2.4 (0.1)	0.2	0.0 to 0.5	0.04
FVC (L) (S.E.)	2.3 (0.1)	2.3 (0.0)	0.1	- 0.0 to 0.3	0.44
FEF_25–75%_ (L/s) (S.E.)	2.7 (0.2)	2.1 (0.1)	0.6	0.2 to 1.0	0.005
Female	n = 7	n = 45			
FEV_1_ (L) (S.E.)	1.4 (0.1)	1.3 (0.1)	0.1	- 0.2 to 0.4	0.43
FVC (L) (S.E.)	3.0 (0.1)	3.1 0.1)	- 0.1	- 0.4 to 0.2	0.67
FEF_25–75%_ (L/s) (S.E.)	1.3 (0.2)	1.3 (0.1)	0.1	- 0.5 to 0.6	0.86

Notes:

General Linear Model (GLM) for remission of asthma to persistent asthma. Estimated marginal means used for determination of height adjusted means.

(n) represents number of participants that provided information.

FEV_1_ = Forced expiratory volume in first second in liters (L) with standard error (S.E.).

FVC = Forced vital capacity in liters (L) with standard error (S.E.).

FEF_25–75%_ = Forced expiratory flow 25 to 75% in liters per second (L/s) with standard error (S.E.).
  At age of 18-years: Children with asthma remission continued to have significantly lower BHR at age 18 years. As expected their lung function was higher and FeNO lower, as they had now lost all symptoms (Table 3 and Figure 1b).

**Table 3:** **Characteristics of asthma remission and persistent asthma at age 18 years**

	**Asthma remission**	**Persistent asthma**	**p-value**
FEV_1_ (L) Mean (SD)	4.2 (0.7)	3.9 (0.8)	0.04
N =	30	96	
BHR (DRS) Mean (SD)	1.1 (0.2)	1.4 (0.5)	<0.001
N =	33	67	
FeNO (ppb) Mean (SD)	27.2 (28.8)	56.3 (54.5)	0.002
N =	25	60	
Atopy % (n)	40.6 (13)	76.6 (72)	<0.001
N =	32	94	
Sputum data	N = 16	N = 22	
Total cell count Median	31.5	38.0	0.41
(25–75 centiles)	(12.9-40.4)	(11.8-66.0)	
% Epithelial cells Median	5.4	7.6	0.28
(25–75 centiles)	(1.4-11.1)	(3.3-13.8)	
% Neutrophils Median	20.0	11.5	0.09
(25–75 centiles)	(8.3-40.8)	(5.5-24.3)	
% Eosinophils Median	0.3	1.0	0.48
(25–75 centiles)	(0–1.4)	(0-3.9)	
ECP (ng/ml)	35.5	119.5	0.09
(25–75 centiles)	(18.7-229.6)	(65.4-279.6)	

Notes:

FEV_1_ was adjusted for height and sex.

Means were compared using two sample *T* test. Pearsons chi-square test was used to compare differences in proportion. Non-parametric test (Mann–Whitney *U* test) was used to compare medians in the samples with non-normal distribution. Two sided significance was set at p <0.05.
2. Early life and adolescent risk factors:; *to identify factors associated with asthma remission.* Tables 4 and 5 show factors associated with asthma remission at univariate level. When adjusted for other factors, male sex and BHR at age 10 showed independent significance in the predictive model (Table 6). The final model demonstrated significance for gain in small airway function towards asthma remission, in addition to lower use of acetaminophen during adolescence and lack of rhinitis at 18 years (Table 6).

**Table 4:** **Factors for remission of asthma up to age 10 years (Predictive factors)**

	**Asthma remission % (n/N)**	**Persistent asthma % (n/N)**	**Odds ratio**	**95% Confidence interval**	**p-value**
Male	68 (38/56)	53 (66/125)	1.9	1.0 – 3.6	0.06
Low Birth Weight^$^	8 (4/53)	5(6/123)	1.6	0.4 – 5.9	0.49
Pre-term delivery*	2 (1/53)	2 (2/123)	1.1	0.1 – 13.1	0.90
Maternal asthma	21.2 (11/52)	30.6 (37/121)	1.6	0.8 – 3.5	0.14
Paternal asthma	11.5 (6/52)	21.5 (26/121)	2.1	0.8 – 5.4	0.09
Recurrent chest infections up to 2 years of age	37 (15/41)	32 (31/98)	1.2	0.6 – 2.7	0.57
Less than 4 months breast feeding	78 (36/46)	65 (72/111)	2.0	0.9 – 4.4	0.10
Formula feeding in first 4 months of life	83 (38/46)	73 (80/109)	0.6	0.2 – 1.4	0.22
Maternal smoking during pregnancy	27 (15/55)	19 (24/124)	1.6	0.7 – 3.3	0.24
Overweight at 10	16.3 (7/43)	23.9 (26/109)	1.6	0.6 – 4.0	0.21
Atopy at 4	42 (16/38)	58 (54/94)	0.5	0.3 – 1.2	0.11
Atopy at 10	43 (20/47)	70 (73/104)	0.4	0.2 – 0.8	0.01
Wheeze at 1 or 2	46 (19/41)	37 (36/98)	1.5	0.7 – 3.1	0.29
Wheeze at 4	63 (32/51)	60 (64/107)	1.1	0.6 – 2.3	0.72
Wheeze at 10	89 (50/56	88 (110/125)	1.1	0.4 – 3.1	0.80
Asthma at age 4	52 (26/50)	56 (59/106)	0.9	0.4 – 1.7	0.67
Rhinitis at 1or 2	25 (11/44)	19 (19/98)	1.0	0.4 – 2.5	0.95
Rhinitis at age 4	15 (8/53)	17 (17/103)	1.0	0.4 – 2.5	1.00
Rhinitis at age 10	46 (25/55)	53 (64/121)	0.7	0.4 – 1.4	0.36
BHR Positive**	51.3 (20/39)	75.2 (79/105)	0.3	0.1 – 0.7	0.006

General Terms.

% (n/N) percentage determined from number of participants with condition/total number of participants with available information on the variable.

Specific Terms.

^$^Low birth weight: <2.5Kg* Pre-term delivery was defined as delivery of child in less than 37 complete weeks of gestation.

**PC20<8mg/ml.

**Table 5:** **Adolescent factors for remission of asthma**

	**Asthma remission**	**Persistent asthma**	**Odds ratio**	**95% Confidence interval**	**p-value**
Male	68 (38/56)	53 (66/125)	1.9	1.0 – 3.6	0.06
Current or past cigarette smoking by study participant at 18 years^#^^%^(n/N)	43 (22/51)	58 (70/120)	0.5	0.3 – 1.1	0.07
Acetaminophen use/month at 18 years (median) (p)^§^	0 (0, 1)	2 (0, 4)	0.8	0.7 – 1.0	0.04
Atopy at 18	43 (13/30)	75 (73/97)	0.3	0.1 – 0.6	0.002
Rhinitis at age 18	34 (19/56)	74 (92/125)	0.2	0.1 – 0.4	<0.001

General Terms.

^%^(n/N) percentage determined from number of participants with condition/total number of participants with available information on the variable.

^#^This includes cigarette smoking by study participants both current and those who have ever smoked cigarettes at any time in the past.

^§^Number of times acetaminophen used over last 12 months.

^¶^Number of times Non-Steroidal Anti-inflammatory drugs used over last 12 months.

**Table 6:** **Adolescent and childhood factors associated with adolescent asthma remission as compared to asthma persistence (Final model)**

**Factor**	**Odds ratio**	**95% Confidence interval**	**p-value**
**Predictive factors**			
Male sex	0.3	0.1 – 0.7	0.005
Bronchial reactivity at 10 years	0.3	0.1 – 0.9	0.03
**Adolescent factors**			
Number of times acetaminophen used per month	0.4	0.2 – 0.8	0.008
Rhinitis at age 18	0.2	0.1 – 0.6	0.004
Gain in small to mid-sized airway function (FEF_25–75%_) between 10 and 18 years	1.7	1.0 – 2.9	0.04
		-	

**Figure 2:**
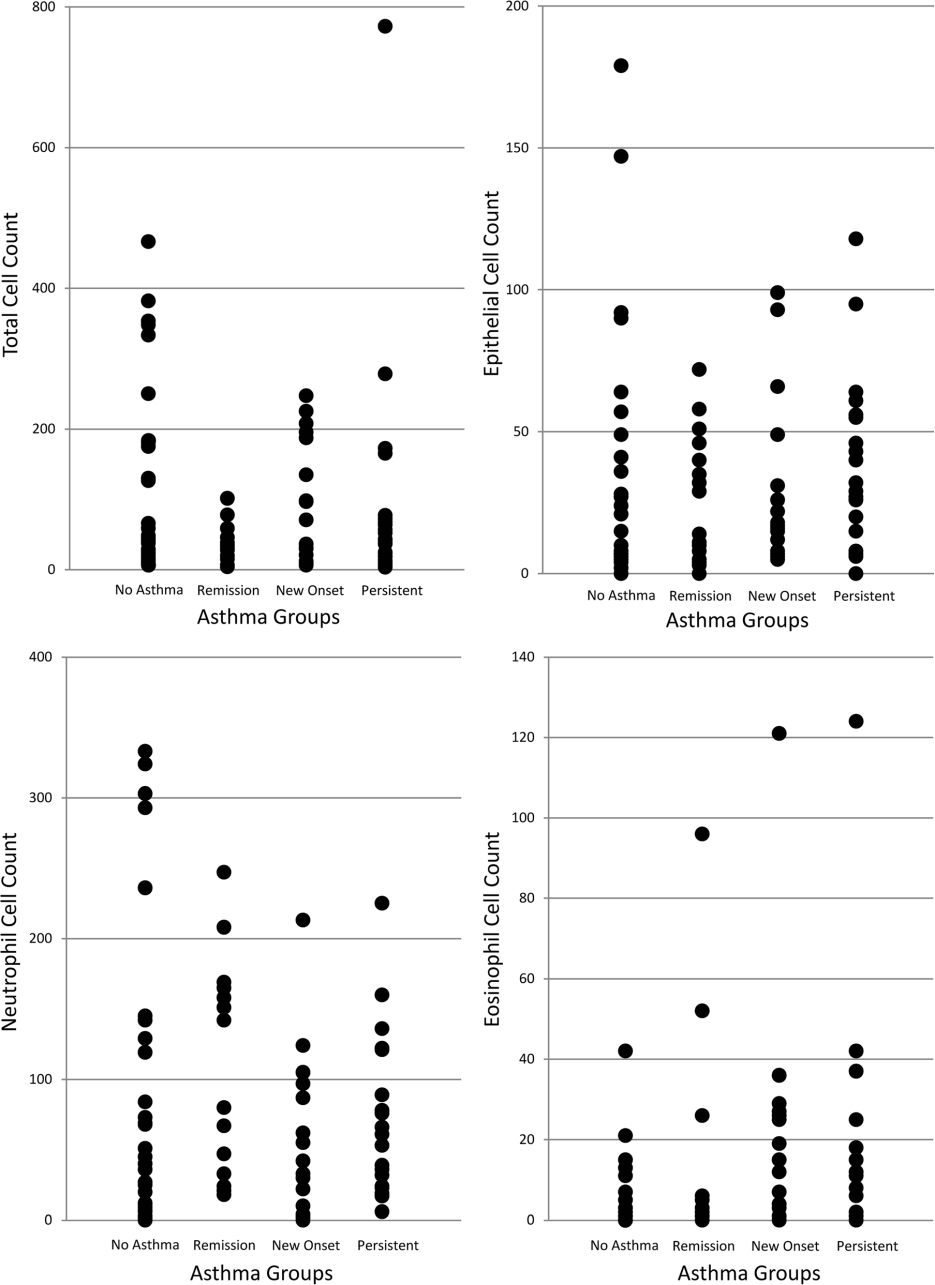
**The total and differential cell counts presented as scatter-plots for various asthma groups.**

Notes:

Odds ratios for remission to persistent asthma are presented by multivariate logistic regression analysis was used with significance determined at p < 0.05.
3. Comparison of asthma remission with never asthma at age 18; *to identify residual pathophysiological abnormalities in remittent asthmatics while symptoms have improved.* We found a low total cell count (median: 31.5, 25–75 centiles: 12.9-40.4) in the sputum of adolescents with asthma remission compared to never asthma (47.0, 19.5-181.3; p = 0.03), even though both groups were asymptomatic at age 18, were not taking inhaled steroids, and their FEV_1_, BHR and FeNO were not significantly different.
4. Comparison of adolescent-onset asthma with persistent asthma; *to investigate the characteristics of asthma that develops during adolescence.* Adolescent-onset asthma demonstrated sputum eosinophilia (3.00%, 0.7-6.6), which was not present in persistent asthma (1.0%, 0–3.9). However, there were no statistically significant differences in physiological or pathological characteristics or in treatment among the two adolescent asthma groups (Figure 2).

### Eosinophils/neutrophils ratio

Adolescent-onset asthma had higher eosinophil count (3.0%, 0.7-6.6) than never asthma (0.3%, 0–1.3); p = 0.005. Among asthma groups, asthma remission had the lowest sputum eosinophil count (0.3%, 0–1.4), similar to never asthma. We used the ratio of sputum eosinophils/neutrophils as a marker of the type of inflammation in different groups. As expected, atopics had a higher ratio (median: 0.1; Interquartile range: 0.8), compared to non-atopic (0.0, 0.1; p = 0.002). Similarly, those with asthma also had a higher ratio (0.2, 0.6), compared to those without at age 18 (0.0, 0.1; p = 0.02). Among asthma groups, the asthma remission had the lowest eosinophils/neutrophils ratio of 0.0 (0.1). Adolescent-onset asthma had a significantly higher eosinophils/neutrophils ratio (0.3, 0.8) compared to never asthma (0.0, 0.1; p = 0.004) and a trend to be high when compared to persistent asthma (0.1, 0.4; p = 0.09).

## Discussion

Adolescents reporting remission of their asthma by age 18 had less BHR at 10 and demonstrated a higher gain in airway function during adolescence compared to persistent asthmatics. They also showed low FeNO and cellular paucity in their airways at age 18, especially of eosinophils with the lowest eosinophil/neutrophil ratio among all groups, including never asthma. Lower use of acetaminophen was the only environmental factor associated with remission amongst multiple factors assessed from birth to adolescence. The adolescent-onset asthma showed prominent eosinophilic inflammation.

The strengths of our study include prospective follow-up from birth, extensive phenotyping including standardized questionnaires on asthma morbidity and treatment, and objective assessments of atopy, lung function and airway inflammation. Thus, our reference group of never asthma had no asthma or wheeze since birth, avoiding misclassification in diagnosis. There are some limitations to this study, such as the relatively small number of subjects who had sputum induction. As a result, some comparison lacked adequate power and may have resulted in false negatives statistics. For example, the subgroup who underwent sputum induction was disproportionately male with higher atopy and FeNO levels. Further, adolescent-onset asthma had a 3-fold higher sputum eosinophilia than persistent asthma, or ECP was more than 3 fold higher in persistent asthma compared to asthma remission but neither of these reached statistical significance. Thus, we suggest that sputum findings should be treated with caution. We also did not have bronchial biopsy or peripheral blood differential counts to compare to the pattern of inflammatory cells seen in sputum.

It is widely acknowledged that some patients with clinical remission of asthma do have persistent BHR and airway inflammation [2,8,9,23]. This is important to recognize in order to understand the biological process behind remission and to estimate the risk of recurrence in future. Some investigators have used the term “complete remission” to identify the sub-group of clinically remittent asthma who may not have BHR or airways obstruction [5]. However, it is hard to define “complete” as this may depend on how extensively one looks for signs of persistent disease. For example, airway inflammation and increased basement membrane thickness was found in those with “complete remission” defined as clinical remission plus lack of BHR [24]. We studied clinical remission from a longitudinal perspective to identify its risk and predictive factors and pathophysiological characteristics. In our study, those with clinical remission had the lowest inflammatory cell counts but they were not different from adolescents with never asthma in terms of lung function, BHR or FeNO, thus seem to have near “complete remission”. Additional questions on the age at which asthma stopped revealed that each individual experienced remission for a minimum duration of 3 years at the 18-year follow-up.

Rapid increase in lung function during adolescence coincides with the age at which most adolescents report losing their asthma symptoms [25]. A higher gain in lung function over adolescence is associated with remission in atopic asthmatics [5]. We may have only seen this in males because our baseline measure of airway function was taken at 10 years when many female participants would already have entered their pubertal growth spurt, unlike the male participants. We have extended these lung function findings to investigate other factors that might have an influence and show that the association between lung function and remission is independent of atopy, as well as gender, smoking and early life risk factors (Table 6). We also found a low BHR at age 10 predicting remission. As airway reactivity is inversely associated with airway caliber [26], our observed decrease in BHR in asthma remission with improved airway function over adolescence, suggests a means by which airway growth contributes towards asthma remission. Our findings are consistent with a previous report of childhood BHR predicting adolescent remission of asthma [27]. Overall, less severe disease (in term of symptoms and BHR) at age 10 promotes remission. We also found that taking acetaminophen during adolescence was associated with persistence of asthma. There is considerable epidemiological evidence that supports an association between acetaminophen use in pregnancy and early infancy with asthma in later childhood [28]. Acetaminophen may exert its effect by increasing oxidative damage of the airway epithelium and thus favors persistence. However, the cause and effect relationship is not clear and the possibility of confounding by indication and reverse causation remains a potential explanation of our reported association of lower acetaminophen use with asthma remission.

Sputum eosinophils are considered a hallmark of atopic asthma [29]. Eosinophil counts in induced sputum correlate with asthma severity in both treated and untreated subjects [30]. A sputum eosinophil count of more than 2 or 2.5% is regarded as indicative of eosinophilic asthma [31,32]. This is relevant clinically in terms of treatment responses [32]. Using this cut-off, there was evidence of eosinophilic-derived inflammation in those with adolescent-onset asthma (eosinophils 3%). Surprisingly, evidence of sputum eosinophils was less pronounced in those with persistent asthma (eosinophils 1%) with no significant differences found when compared to never asthma, while eosinophil count was significantly higher in adolescent-onset asthma compared to never asthma (eosinophils 0.3%). Corticosteroid treatment can suppress airway eosinophils, however, we did not find any difference in the proportion taking inhaled corticosteroids in the persistent asthma compared to the adolescent-onset asthma (Table 5). Wasserman et al. in a recent cross-sectional study showed that asthma remission is associated with reduced inflammation with low eosinophil counts and ECP as well as reduction in both T helper (h)1 (interleukin (IL)-12, IFN-γ) and Th2 cytokines (IL-5) [33]. However, contrary to our findings, they did not find differences in total cell count between asthma remission and never asthma. Moreover, they found high eosinophils in the continuing (persistent) asthma groups, while we found sputum eosinophils to be prominent in the adolescent-onset asthma, but this group was not included in their study.

The main feature of the asthma remission was a paucicellular sputum with very low total cell count. This low total cell count (lower than never asthma) could be indicative of an overall low level of airway inflammation and cellular infiltration driving remission of asthma. Specifically, eosinophil counts were very low at 0.3% and the eosinophil/neutrophil ratio in asthma remission (median 0.01) was 3-fold lower than never asthma (median 0.03) and 30-fold lower than adolescent-onset asthma (median 0.3). Further, FeNO, a marker of eosinophilic inflammation, was lower in asthma remission than persistent asthma (Table 3) and same as never asthma (Table 4).

## Conclusions

Asthma remission was associated with less severe disease before adolescence (low symptoms and BHR), male sex, greater gain in small airways function in boys and low airway cellular infiltrate, while asthma that develops during adolescence is primarily eosinophilic. Further studies using bronchial biopsies to correlate findings in sputum with that occurring in the airway wall are required to understand the mechanisms associated with changes in cellular and physiological profiles of asthma phenotypes during adolescence.

## Additional file

Additional file 1:
**Number (%) of participants with data available at 10 and 18 years for various assessments.** Characteristics of adolescents in each group who had sputum induction compared to those who did not. Lung function and airway inflammation in atopic and non-atopic subjects. Lung function and airway inflammation in adolescent asthma at age 18 years. Factors for remission of asthma up to age 10 years (Predictive factors). Adolescent factors for remission of asthma.

## Abbreviations

BHR: Bronchial hyperresponsiveness
DRS: Dose response slope
SPT: Skin prick test
FeNO: Fractional exhaled nitric oxide
FEV_1_: Forced expiratory volume in one second
ECP: Eosinophilic cationic protein
IQR: Interquartile range
OR: Odds ratio
CI: Confidence interval
ATS: American Thoracic Society
