# Supplementary material for: Pathophysiological characterization of asthma transitions across adolescence
Source: Respir Res. 2014 Nov 29;15(1):153. doi: 10.1186/s12931-014-0153-7 (PMC4256730; doi:10.1186/s12931-014-0153-7)
Supplement: Additional file 1: — Number (%) of participants with data available at 10 and 18 years for various assessments. Characteristics of adolescents in each group who had sputum induction compared to those who did not. Lung function and airway inflammation in atopic and non-atopic subjects. Lung function and airway inflammation in adolescent asthma at age 18 years. Factors for remission of asthma up to age 10 years (Predictive factors). Adolescent factors for remission of asthma. [file 12931_2014_153_MOESM1_ESM.docx]

ADDITIONAL TABLES AND FIGURES

**Table S1: Number (%) of participants with data available at 10 and 18 years for various assessments.**

|  | 10 years  (N=1456) | 18 years  (N=1456) |
| --- | --- | --- |
| Questionnaires (all) | 1373 (94.3%) | 1313 (90.2%) |
| Questionnaires (face to face) | 1043 (76.0) | 864 (65.8%) |
| Questionnaires (telephone/postal) | 330 (24.0) | 449 (34.2%) |
| Skin Prick Test | 1036 (71.2%) | 855 (58.7%) |
| Spirometry | 981 (67.4%) | 840 (57.7%) |
| Bronchial challenge | 784 (53.9%) | 587 (40.3%) |
| Exhaled Fractionated Nitric Oxide | _ | 822 (56.5%) |
| Sputum | _ | 100 (6.9%) |

Table S2: Characteristics of adolescents in each group who had sputum induction compared to those

who did not.

|  | Never asthma | | Asthma remission | | Adolescent- onset asthma | | Persistent asthma | |
| --- | --- | --- | --- | --- | --- | --- | --- | --- |
|  | All  (n=959) | Sputum  (n=33) | All (n=56) | Sputum (n=18) | All (n=95) | Sputum (n=20) | All (n=125) | Sputum (n=29) |
| Sex (male) %  (n) | 49.4  (474) | 60.6  (20) | 67.9  (38) | 77.8  (14) | 37.9  (36) | 60.0  (12) | 52.8  (66) | 55.2  (16) |
| Height mean, m  (SD) | 171.2  (9.4) | 174.3  (8.8) | 172.5  (9.8) | 176.3  (10.6) | 168.8  (11.6) | 171.2  (9.8) | 171.8  (9.3) | 171.8  (10.4) |
| Weight mean, kg  (SD) | 67.4  (13.0) | 66.0  (11.2) | 67.7  (13.5) | 68.5  (13.2) | 69.1  (15.7) | 65.8  (11.2) | 70.5  (16.3) | 70.7  (17.1) |
| Atopy %  (n/N) | 33.8  (215/636) | 42.4  (14/33) | 43.3  (13/30) | 50.0  (9/18) | 58.5  (38/65) | 75.0  (15/20) | 75.3  (73/97) | 72.4  (21/29) |
| Rhinitis %  (n/N) | 28.8  (276/957) | 12.1  (4/33) | 34.0  (19/56) | 16.7  (3/18) | 57.9  (55/95) | 65.0  (13/20) | 73.6  (92/125) | 65.6  (19/29) |
| Eczema %  (n/N) | 11.1  (106/957) | 6.1  (2/33) | 12.5  (7/56) | 16.7  (3/18) | 11.6  (11/95) | 10.0  (2/20) | 20.2  (25/124) | 20.7  (6/29) |
| FEV_1_ mean, L  (SD) | 4.1  (0.8) | 4.5*  (0.9) | 4.2  (0.8) | 4.2    (0.7) | 3.8  (0.9) | 3.8  (0.8) | 3.8  (0.8) | 4.0  (0.8) |
| BHR mean  (SD) | 1.1  (0.2) | 1.0  (0.1) | 1.1  (0.2) | 1.0  (0.2) | 1.3  (0.4) | 1.3  (0.4) | 1.4  (0.5) | 1.4  (0.5) |
| FeNO mean, ppb  (SD) | 22.2  (22.4) | 22.2  (12.6) | 27.5  (29.4) | 30.1  (30.2) | 41.1  (41.8) | 68.7  (57.3) | 55.7  (54.2) | 60.4  (48.8) |

Notes:

*Significant at p=0.002. All other comparisons were non-significant.

Categorical factors analyzed using Chi square test or Fisher`s exact test (when <5 in a group).

Continuous variables analyzed using independent sample T test.

**Table S3: Lung function and airway inflammation in atopic and non-atopic subjects**

|  | **Non-atopic** | **Atopic** | **P-value** |
| --- | --- | --- | --- |
|  | N=41 | N=59 |  |
| FEV_1_ (L)  Mean (SD) | 4.2 (0.9) | 4.2 (0.8) | 1.00 |
| *BHR (DRS)  Mean (SD) | 1.0 (0.1) | 1.3 (0.4) | <0.001 |
| FeNO  Mean (SD) | 17.4 (8.4) | 56.0 (47.1) | <0.001 |
| Current asthma % (n) | 31.7 (13) | 61.0 (36) | 0.003 |
| Inhaled steroids | 14.8 (4/27) | 54.5 (24/44) | 0.001 |
| Sputum data | N=38 | N=48 |  |
| Total cell count Median  (25-75 centiles) | 38.5  (18.8-74.5) | 34.5  (13.0-130.0) | 0.79 |
| % Epithelial cells Median  (25-75 centiles) | 3.1  (1.5-10.1) | 6.9  (2.8-14.4) | 0.02 |
| % Neutrophils Median  (25-75 centiles) | 12.0  (4.9-34.1) | 14.5  (5.0-30.8) | 1.00 |
| % Eosinophils Median  (25-75 centiles) | 0.3  (0-0.8) | 1.7  (0.3-6.0) | <0.001 |
| ECP (ng/ml) | 91.7  (38.1-225.6) | 95.3  (34.9-292.2) | 0.63 |

Notes: The number refer only to subjects with sputum samples

*Dose response curve (DRS) is a continuous measure of bronchial hyperresponsiveness (BHR).

FEV_1_ was adjusted for height and sex.

Data were collected at age 18 years. Cellular data are from sputum samples.

Means compared with two sample t test;

Medians compared using non-parametric (Mann-Whitney U) test.

Table S4: Lung function and airway inflammation in adolescent asthma at age 18 years

|  | No-asthma | Asthma | p-value |
| --- | --- | --- | --- |
|  | N=51 | N=49 |  |
| FEV_1_ (L) Mean (SD) | 4.4 (0.8) | 3.9 (0.8) | 0.002 |
| *BHR (DRS) Mean (SD) | 1.0 (0.1) | 1.4 (0.4) | <0.001 |
| FeNO (ppb) Mean (SD) | 24.9 (20.2) | 63.9 (51.6) | 0.001 |
| Atopy % (n) | 45.1 (23) | 73.5 (36) | 0.003 |
| Sputum data | N=46 | N=40 |  |
| Total cell count  Median  (25-75 centiles) | 38.0  (17.5-101.5) | 38.0  (12.0-97.5) | 0.81 |
| % Epithelial cells Median  (25-75 centiles) | 4.5  (1.5-11.0) | 6.5  (2.3-13.4) | 0.16 |
| % Neutrophils Median  (25-75 centiles) | 16.9  (5.2-39.5) | 10.2  (4.6-22.1) | 0.12 |
| % Eosinophills Median  (25-75 centiles) | 0.3  (0-1.3) | 1.7  (0.3-5.9) | 0.02 |
| ECP (ng/ml) | 62.3  (25.4-229.3) | 115.0  (62.1-375.0) | <0.05 |

Notes: The number refer only to subjects with sputum samples

*Dose response curve (DRS) is a continuous measure of bronchial hyperresponsiveness (BHR).

FEV_1_ was adjusted for height and sex.

Data were collected at age 18 years. Cellular data are from sputum samples.

The “No-asthma” category includes participants from the “never” and “remission” asthma groups.

The “Asthma” category includes participants from the “Adolescent-onset” and “persistent” asthma groups.

Means compared with two sample t test;

Medians compared using non-parametric (Mann-Whitney U) test.
